# Innate immunity of bile and cholangiocytes in primary biliary cholangitis

**DOI:** 10.3389/fimmu.2025.1655287

**Published:** 2025-10-02

**Authors:** Ran Chen, Yan Sun, Ying Hu, Wenlin Tai

**Affiliations:** ^1^ Clinical Laboratory, The Second Affiliated Hospital of Kunming Medical University, Kunming, Yunnan, China; ^2^ Pharmaceutical College and Key Laboratory of Pharmacology for Natural Products of Yunnan Province, Kunming Medical University, Kunming, Yunnan, China; ^3^ College of Modern Biomedical Industry, Kunming Medical University, Kunming, Yunnan, China

**Keywords:** primary biliary cholangitis (PBC), innate immunity, biliary tract, cholangiocyte, therapeutic target, prognostic assessment

## Abstract

Primary Biliary Cholangitis (PBC) is a chronic autoimmune liver disease characterized by immune-mediated destruction of intrahepatic bile ducts. This review synthesizes current knowledge on the critical role of innate immunity, specifically involving cholangiocytes, bile components, and associated immune cells. Cholangiocytes function not only as passive targets but also as active immunomodulators through mechanisms including Toll-like receptor (TLR) signaling, antigen presentation, and immune cell recruitment. Dysregulated bile acid signaling via receptors like TGR5 disrupts immune homeostasis, while apoptosis of biliary epithelial cells releases antigens (e.g., PDC-E2), triggering aberrant innate and adaptive immune responses. Innate lymphoid cells (ILCs), natural killer (NK) cells, and macrophages exhibit altered frequencies and functions in PBC, driving chronic inflammation and fibrosis through cytokine cascades (e.g., IL-17, IFNγ) and interactions within the gut-liver axis. Furthermore, biliary microbiota dysbiosis exacerbates disease by promoting bacterial translocation, modifying bile acid metabolism, and activating innate immune pathways. Current clinical management with ursodeoxycholic acid (UDCA) and obeticholic acid (OCA) primarily addresses cholestasis. However, the immunomodulatory effects of these agents remain constrained. Targeted therapeutic strategies addressing innate immune pathways—exemplified by RIPK2 (Receptor Interacting Serine/Threonine Kinase 2) inhibition, IL-1 blockade(Canakinumab), and T cell immunoglobulin mucin domain-containing protein 3 (TIM-3) modulation—alongside cell-based interventions such as mesenchymal stem cell therapy, demonstrate considerable therapeutic potential. Advancing these modalities necessitates multidisciplinary integration to facilitate clinical translation. Additionally, Prognostic indices like the neutrophil-to-lymphocyte ratio (NLR) and monocyte-to-lymphocyte ratio (MLR) reflect systemic inflammation and correlate with disease progression. Achieving therapeutic precision requires deeper elucidation of the gut-biliary-immune axis, trained immunity mechanisms, and cholangiocyte senescence, paving the way for targeted interventions in PBC. Establishing a comprehensive treatment burden assessment system is imperative to facilitate the transition from investigational platforms to clinical care.

## 1 Introduction

Primary Biliary Cholangitis (PBC) is a chronic autoimmune disorder characterized by immune-mediated destruction of interlobular and septal bile ducts. Left untreated, this pathological trajectory evolves through progressive cholestasis and chronic inflammation, culminating in irreversible hepatic fibrosis and cirrhosis via sustained fibrogenesis (1, 2). The disease pathogenesis entails multifactorial interactions among genetic predisposition (notably HLA class II alleles), epigenetic alterations, and environmental triggers, collectively initiating autoimmune targeting of biliary epithelial cells (1, 2). Diagnostic classification for PBC includes three key parameters: AMA status, histopathological staging (Ludwig or Nakanishi), and clinical phenotype (3–5). AMA sensitivity is 90-95%, but AMA-negative cases require autotaxin levels and cholangiographic imaging for confirmation (5–7). First-line treatment is UDCA at 13–15 mg/kg/day, achieving biochemical response in 60-70% of patients via bile acid modulation and anti-apoptotic mechanisms (8, 9). For UDCA non-responders (40% of cases), second-line agents like obeticholic acid and PPAR agonists are implemented to impede progression (9). Post-liver transplant patients with relapse and poor UDCA response exhibit elevated mortality risk (10). Clinical trials should identify and validate surrogate markers as endpoints for evaluating second-line therapies (11, 12). The Ursodeoxycholic Acid Response Score (URS) may serve as a predictive tool for long-term clinical outcomes—such as liver transplantation or death—following 12 months of pre-therapeutic UDCA administration (13, 14). Specifically, the GLOBE and UK-PBC Risk Scores guide second-line therapy allocation in practice (15). High-risk patients can be prioritized for prompt second-line treatments such as obeticholic acid (16), while Paris II criteria offer standardized patient stratification for trials (17). With individualized medicine advancing, models integrating multifactorial data are essential for optimizing PBC management (18). Although well-validated prognostic models are applicable to all patients with PBC, their clinical utility in guiding therapy is most pronounced in individuals responsive to UDCA. For up to 40% of patients who do not respond to UDCA as first - line therapy, and considering that a substantial proportion may also experience failure of second - line treatment options, the development of therapeutic strategies remains an unmet clinical requirement.

Autoimmune bile duct lesions may arise following disruption of tolerance mechanisms mediated by bile duct epithelial cells (19). Biliary epithelial cells establish a sophisticated interactive network with diverse hepatic immune cell populations, facilitating leukocyte recruitment to specific anatomical sites through cytokine and chemokine expression (20). In cholangiopathies, immune cells—including monocytes, lymphocytes, neutrophils, and mast cells—are recruited to the liver. Within this microenvironment, they interact with biliary epithelial cells and resident Kupffer cells, collectively influencing disease progression (21).

Crucially, interactions between biliary epithelial cells and immune cells are pivotal for maintaining homeostasis. Additionally, biliary epithelial cells can mitigate cholangitis development through upregulation of PD-L1 expression, thereby conferring protection against CD8^+^T cell-mediated cytotoxicity (22). During PBC pathogenesis, Damage-associated Molecular Patterns (DAMPs) released during biliary epithelial cell apoptosis trigger innate immune responses via activation of Pattern Recognition Receptors (PRRs), including TLRs and NOD-like receptors (NLRs) (23). These DAMPs encompass autoantigens such as the mitochondrial Antigen Pyruvate Dehydrogenase Complex- E2 (PDC-E2), whose aberrant exposure disrupts normal immune tolerance (24). Disordered bile acid metabolism potentiates DAMP release, establishing a pathological positive feedback loop (25). Activated PRRs induce chemokine secretion through MyD88-dependent signaling pathways, promoting infiltration of innate immune cells—including Ly6C^+^monocytes, macrophages, and Dendritic Cells (DCs) (26). These cells subsequently establish the foundation for adaptive immune responses by presenting PDC-E2 antigen and secreting pro-inflammatory cytokines (such as IL-12, IL-23) (24). Metabolites generated during intestinal dysbiosis, such as short-chain fatty acids, can further perturb Myeloid-derived Suppressor Cell (MDSC) homeostasis, exacerbating innate immune dysregulation (27). The innate-adaptive immune crosstalk constitutes a core immunological principle, facilitating synergistic amplification of the immune response. Autoantigens presented by innate immune cells activate CD4^+^T cells, specifically the CCR5^+^CD4^+^T cell subset (28). IL-15Rα^+^B cells promote activation and expansion of these T cells via IL-15 signaling, orchestrating targeted immune attacks against the biliary epithelium (28).This paradigm emphasizes that innate immunity functions not merely as an initiator but also as a critical modulator of adaptive immunity, thereby mediating dynamic cross-regulation between these two systems.

AMA is the most classic hallmark, but other antibodies such as elevated serum IgM and specific antinuclear antibodies also play critical roles in diagnosis and prognosis. Non-AMA autoantibodies, particularly specific subtypes of antinuclear antibodies (ANA), including anti-gp210, anti-sp100, and anti-centromere, constitute an essential component of PBC diagnosis. They are especially important in cases where AMA is negative (3–5). Immunomodulation constitutes a systemic therapeutic approach. Despite the presence of characteristic AMA-M2, current immunomodulatory strategies still lack cellular specificity and precise targeting. Furthermore, the translational relevance of animal models remains debatable, as they fail to accurately identify therapeutic windows or adequately replicate the spatiotemporal heterogeneity of the immune microenvironment. Achieving therapeutic efficacy necessitates maintaining a delicate balance—a critical consideration warranting in-depth investigation.

**Figure 1:**
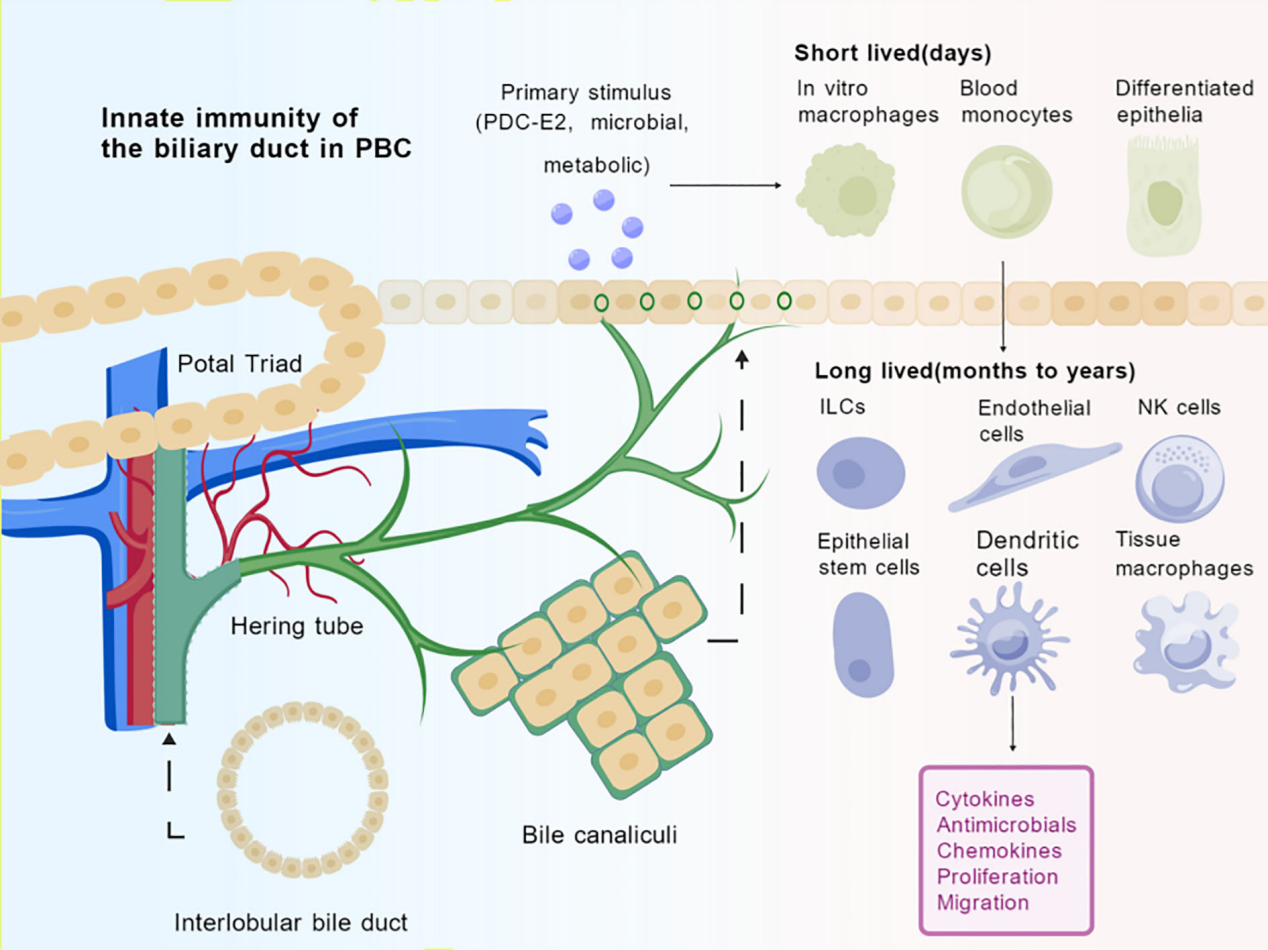
Innate immune cells in the bile duct environment of PBC. Spatial arrangements and dynamic transformations of cells in biliary duct transport compartments, organized around Hering tube and bile canaliculi to form a conduit for immune cell trafficking. The initial immunogenic insult (e.g., biliary epithelial exposure to PDC-E2, microbial products, or metabolic stressors) induces the recruitment of short-lived innate immune cells (e.g., monocytes, macrophages) from peripheral circulation. Within the tissue microenvironment, a complex interplay takes place among long-lived tissue-resident cells (e.g., macrophages, NK cells, ILCs, dendritic cells), cholangiocytes, and endothelial cells. The sustained release of pro-inflammatory cytokines, chemokines, antimicrobial peptides, aberrant proliferation, and migratory responses, culminating in the progressive autoimmune targeting of interlobular bile ducts. Created by BioGDP.com (29). PDC-E2, Pyruvate Dehydrogenase E2; ILCs, Innate lymphoid cells, NK cells, Natural Killer cells.

Therefore, this review provides an overview of biliary tract innate immunity, focusing on cholangiocytes, bile constituents, and immune cells in PBC from an immunological perspective (as illustrated in **Figure 1**).

We aim to concentrate on the role of innate immune imbalance in PBC pathogenesis and how addressing these mechanisms might offer new therapeutic possibilities. The first-line therapy for PBC remains UDCA. Based on validated prognostic models such as the URS, GLOBE, and UK-PBC scores, patients with high-risk PBC are prioritized candidates for second-line therapies, these established prognostic markers and treatment strategies are supported by robust clinical evidence and recommended for use in international guidelines. However, it is worth noting that emerging therapeutic approaches including RIPK2 inhibition, IL-1 blockade, TIM-3 modulation, and MSCs therapy are currently given the involvement of innate immunity in PBC pathogenesis. Prognostic indices such as NLR and MLR reflect systemic inflammation and correlate with disease progression. Moving forward, achieving therapeutic precision will require deeper mechanistic insights into the gut-biliary-immune axis, trained immunity, and cholangiocyte senescence to enable targeted interventions. Nevertheless, it must be mentioned that these approaches are currently primarily at the experimental stage, and their clinical translation necessitates rigorous validation through further clinical trials to ensure both safety and efficacy as potential innate immunomodulatory interventions.

## 2 The innate immune system of the biliary tract

### 2.1 Cell composition, function and pathways

**Figure 2:**
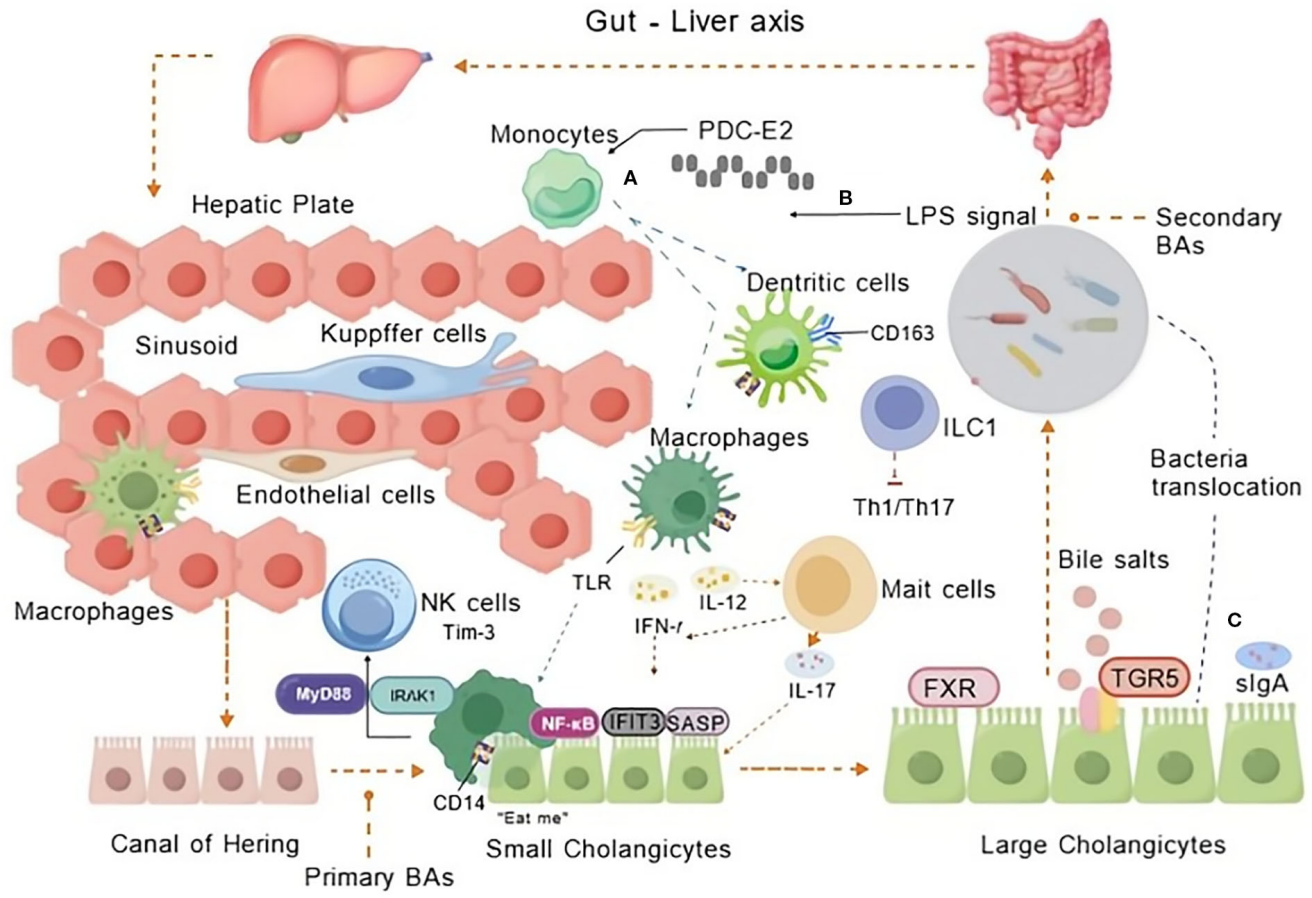
Innate immune cells in PBC. The interplay of innate immune cells in PBC’s hepatic sinusoids and biliary system with micro-organisms traversing hepatoenteric circulation during BA metabolism. Innate immune mechanisms via the gut-liver axis contribute to PBC pathogenesis through gut-derived signals and hepatic inflammation. **(A)** Monocyte recruitment and hepatic activation: Monocytes infiltrate the liver sinusoids and interact with endothelial cells, Kupffer cells, and macrophages. Mitochondrial antigen PDC-E2 triggers monocyte recruitment and activation via NF-κB signaling (through CD14/TLR4), alongside regulatory interactions with NK cells and immune checkpoint regulator TIM-3 modulate cytotoxicity. **(B)** LPS-induced inflammation: LPS translocated from the gut activates TLR signaling on innate immune cells (e.g., macrophages, dendritic cells), promoting pro-inflammatory cytokine production (e.g., IL-12, IL-23, IL-17, IFN-γ). **(C)** Bacterial translocation and bile acid dysregulation: sIgA function and gut barrier failure lead to bacterial translocation. Secondary BAs activate TGR5 receptors, disrupting BA homeostasis and amplifying inflammatory and cholestatic injury in small and large cholangiocytes. Created by BioGDP.com (29). BAs, Bile Acids; TLR, Toll-like receptor; LPS, Lipopolysaccharide; MyD88, Myeloid differentiation factor 88; IRAK1, Immune Recognition of Alphavirus Capsid Protein; IFIT3, Interferon-induced protein with tetratricopeptide repeats 3; SASP, Senescence-Associated Secretory Phenotype; TIM-3, an immune checkpoint molecule; FXR, Farnesoid X Receptor; TGR5, Takeda G protein-coupled receptor 5; ILC1, Innate Lymphoid Cells; CD163, a marker for macrophages; sIgA, Secretory Immunoglobulin A.

The biliary tract orchestrates innate immunity through coordinated interactions between its cellular components, biochemical mediators, and microbial residents(as shown in **Figure 2**).

As the primary defense mechanism, macrophages, dendritic cells, and innate lymphoid cells strategically localize within portal tracts and bile ducts, establishing surveillance networks that detect gastrointestinal-derived pathogens via pattern recognition receptors (30). These immune effectors collaborate with cholangiocytes—transcending their structural role—which actively modulate immune responses through cytokine secretion, antigen presentation, and secretory IgA production to neutralize invading microorganisms (31). Significantly, this cellular interplay is biochemically regulated by Bile Acids(BAs) via TGR5 receptor signaling on cholangiocytes. BA-mediated induction of anti-inflammatory mediators counterbalances pro-inflammatory signals to maintain mucosal homeostasis. The system’s resilience further relies on bidirectional crosstalk with the biliary microbiota: commensal organisms metabolize BAs into immunoregulatory derivatives, while dysbiosis disrupts this equilibrium, predisposing to cholangiopathies such as primary biliary cholangitis through aberrant immune activation (32, 33). Notably, cholangiocyte viability constitutes a critical determinant—their senescence impairs antimicrobial defense, whereas apoptosis initiates fibrotic cascades, thereby delineating how epithelial-immune coordination governs disease progression (18). Consequently, emerging therapeutic strategies target this multifaceted network, aiming to recalibrate BA signaling, restore microbial symbiosis, and preserve cholangiocyte function to intercept pathological cascades.

The innate immune signaling pathways within the biliary tract encompass complex molecular mechanisms regulating host responses to microbial stimuli. While direct investigations of the biliary tract remain limited, insights derived from related tissues and cell types provide valuable insights into these pathways. NF-κB signaling constitutes a pivotal component of innate immune responses across diverse epithelial tissues. NF-κB activation is essential for epithelial defense, with cortactin playing a critical role in facilitating NF-κB-mediated cytokine production, such as IL-8, during bacterial infection (32). This underscores the significance of NF-κB in mediating inflammatory responses, likely relevant to biliary epithelial cells due to their epithelial characteristics. Similarly, microbial components modulate innate immune signaling, as demonstrated in studies of pathogen recognition. *Chlamydia trachomatis* can induce TLR3 expression while concurrently downregulating the NF-κB and IRF3 pathways in Sertoli cells, resulting in the suppression of pro-inflammatory cytokine production (33). This indicates that certain pathogens can manipulate innate signaling pathways, potentially influencing immune responses in tissues such as the biliary tract that are exposed to microbial stimuli.

Type I and III interferons play critical roles in innate antiviral immunity. Studies demonstrate differential interferon responses in human nasal versus lung tissues following SARS-CoV-2 infection, revealing restricted interferon induction in lung tissue despite productive viral infection (34, 35). These findings indicate tissue-specific regulation of interferon signaling pathways, suggesting parallels to the response mechanisms observed in biliary epithelium upon exposure to pathogens. The role of cytokines, such as Interferon Gamma (IFNγ), in linking immune responses to tissue pathology is underscored (36), who found that chronic IFNγ expression alters hepatic immune microenvironments, potentially contributing to autoimmune conditions like primary biliary cholangitis.

This connection underscores the critical role of cytokine signaling pathways in biliary immune regulation. Furthermore, the involvement of nuclear receptors in modulating immune responses is evident within the context of liver and biliary diseases. Studies indicate that PPARα and FXR may alter the hepatic immune microenvironment in biliary atresia (37, 38), suggesting that nuclear receptor pathways are integral to immune regulation in biliary tissues. Additional insights into innate immune regulation derive from investigations of tissue-specific responses. Bacterial outer membrane proteins activate innate immunity via neural-immune communication pathways, demonstrating that microbial components may influence immune signaling beyond classical receptor pathways (39). Moreover, the multifunctional role of CD14 in innate immunity and tissue homeostasis has highlighted its potential regulatory functions in barrier tissues characterized by rapid cell turnover, such as the biliary epithelium (40).

### 2.2 The relationship between innate biliary immunity and PBC

Typically, Biliary epithelial cells (BECs) are integral to immune response, functioning not only as a physical barrier but also actively participating in immune signaling. The apoptosis of BECs induces the release of auto-antigenic epitopes, which subsequently activate the immune system, driving dysregulation of both innate and adaptive immunity in PBC (41). Mass cytometry analyses demonstrate quantitative alterations in peripheral immune cell subsets in patients, including decreased γδ T cells and memory B cells, alongside increased monocytes and naïve B cells (42). This cellular imbalance underscores the critical role of innate immunity in disease progression (43).

Cytokines and immune mediators orchestrate PBC pathogenesis. Elevated expression of interferon-induced proteins, such as IFIT3, within senescent BECs indicates their involvement in inflammatory processes (44). Furthermore, TIM-3-mediated modulation of chemokine receptors on NK cells contributes to immune dysregulation, suggesting therapeutic potential through targeting this pathway (45). The gut microbiome exhibits significant interaction with innate immunity in PBC. Disease-associated dysbiosis, characterized by reduced microbial diversity and overgrowth of specific bacterial taxa, may influence immune responses via the gut-liver axis (46). Microbial metabolites can modulate both innate and adaptive immunity, thereby linking intestinal homeostasis to biliary inflammation (47).

Identification of disease-associated immune cell subsets and cytokine profiles holds promise for yielding novel biomarkers and therapeutic targets. Promising strategies include modulating the TIM-3 pathway and restoring gut microbiome balance (45, 46). Concurrently, genome-wide association studies identify risk loci predominantly related to immune function, highlighting the contribution of innate immunity to genetic susceptibility in PBC (43).

## 3 The mechanism of onset and progression

### 3.1 The autoimmune response

The innate immune system plays a critical role in the pathogenesis of PBC. NK cells mediate the destruction of BECs through both direct and indirect mechanisms. Specifically, the enhanced frequency and cytotoxicity of NK cells observed in the peripheral blood and liver tissues of PBC patients amplify autoimmune responses via the activation of autoreactive CD4+ T cells and the secretion of inflammatory cytokines (48). Innate Lymphoid Cells (ILCs) exhibit imbalances in subtype distribution among PBC patients, with alterations in cytokine production patterns correlating with disease severity. These ILC dysregulations may promote inflammatory and autoimmune pathways during PBC progression (49). Emerging evidence highlights trained immunity—a persistent functional reprogramming of innate immune cells following initial stimuli—as a contributor to exaggerated inflammatory responses upon secondary challenges (50). This innate immune hyper-reactivity to self-antigens may perpetuate chronic inflammation and tissue injury. The metabolic and epigenetic remodeling underlying trained immunity represents potential therapeutic targets. Reversing these adaptations may attenuate PBC-associated chronic inflammation (51). Pro-inflammatory cytokines secreted by activated innate immune cells establish a destructive microenvironment for BECs. Notably, the Th17/Treg imbalance, characterized by elevated Th17 cell levels and reduced Treg cell levels, reflects the inflammatory shift characteristic of PBC (52).

Gut microbiome dysbiosis in PBC patients correlates with altered microbial diversity and metabolite profiles. Microbiota-derived metabolites (e.g., short-chain fatty acids) modulate innate immunity, suggesting microbiome-targeted therapies could influence disease progression (46). Furthermore, metabolites derived from the gut microbiota reach the liver via the portal venous system, providing persistent low-grade immune stimulation. This continuous gut-derived immunogenic signal is hypothesized to disrupt immune tolerance towards mitochondrial antigens (53). Consequently, this ongoing microbial-driven immune activation provides a mechanistic explanation for the suboptimal efficacy and lack of durability observed with therapeutic strategies solely targeting systemic immune responses (53). Concurrently, the pivotal role of Peroxisome Proliferator-Activated Receptor (PPAR) in regulating the gut-liver immune axis has emerged as a significant finding (23). PPAR agonists to modulate immune responses, Low-dose IL-2 to restore immune balance (52), Trained immunity pathway inhibitors (50, 54). Their involvement in modulating immune and metabolic pathways offers promising novel targets for therapeutic intervention in PBC.

### 3.2 Injury of bile duct epithelial cells

Biliary epithelial cells are primary targets in PBC, where apoptosis releases autoantigen epitopes that trigger immune activation. This process is modulated by genetic predisposition and environmental factors that disrupt immune tolerance, driving disease progression (41). Bile acid accumulation within BECs induces cellular damage through membrane-disrupting detergent effects (55). Concurrently, diminished bicarbonate production compromises the protective “bicarbonate umbrella,” worsening BEC injury and cholestasis (56). Innate immunity critically influences PBC pathogenesis. Inflammatory cytokines released during BEC injury recruit and activate macrophages and NK cells, amplifying bile duct damage. Invariant NK T cells exacerbate liver fibrosis via Interleukin-17A (IL-17A) production, correlating with disease severity in PBC patients (56). Mast cells further contribute by interacting with innate immune cells to promote inflammation and fibrosis in cholestatic liver diseases (57).

Cholangiocytes exhibit injury responses including proliferation, differentiation, and senescence, collectively termed the Ductular Reaction (DR) to repair bile ducts (58). However, maladaptive responses exacerbate fibrosis. Senescent cholangiocytes adopt a Senescence-associated Secretory Phenotype (SASP), releasing pro-inflammatory mediators that sustain fibrogenesis and perpetuate injury cycles (44). Advancements in organoid technology have enabled the modeling of BEC injury, providing insights into cholangiocyte pathophysiology. These model systems facilitate the investigation of cholangiocyte apoptosis and fibrogenic responses critical to the progression of cholangiopathies, including biliary atresia and PBC (59). Such models hold promise for identifying therapeutic targets to mitigate BEC damage in PBC.

UDCA targets enhanced bile flow and reduced cholestatic injury but frequently fails to halt disease progression (60). Emerging therapeutic strategies focus on immunomodulatory agents and anti-fibrotic therapies to improve BEC survival and restore biliary function (55). Targeting cholangiokines—cytokines secreted by cholangiocytes—may also modulate the hepatic microenvironment for therapeutic benefit (61). The complex interplay between BEC injury, immune activation, and cholangiocyte adaptive responses underscores the disease pathogenesis.

### 3.3 Inflammatory factors

Inflammatory factors—ranging from molecular signaling pathways like TGF-β1/Smad and IRF3 phosphorylation, to immune cell subsets such as ILCs, and systemic cytokines—play crucial roles in the innate immune response of the biliary tract in PBC. These factors contribute to inflammation, tissue injury, and fibrosis, shaping the disease course and offering potential targets for therapeutic intervention.

MicroRNA-34a has been identified as a potential marker and regulator of fibrogenesis in PBC which promotes Epithelial-Mesenchymal Transition (EMT) and liver fibrosis by modulating the TGF-β1/Smad pathway, suggesting its pivotal role in inflammatory and fibrotic processes within the biliary system (62). However, the precise role of EMT in the pathogenesis of PBC remains controversial. Although *in vitro* studies demonstrate that TGF-β1 can induce EMT in cholangiocytes (63, 64), lineage-tracing animal models have failed to provide definitive evidence for EMT occurrence *in vivo* (65, 66). Cholangiocyte senescence (67, 68), autophagy dysfunction (69), and inflammatory cytokines such as IL-17A (70) are recognized as significant pathogenic mechanisms in PBC. As a matter of fact, whether EMT acts as a primary driver of bile duct injury or a secondary consequence requires further investigation. The extent of concordance between EMT manifestations observed in human PBC and those in experimental animal models also warrants critical evaluation.

Moreover, substantial evidence suggests that interferon regulatory factor 3 (IRF3) phosphorylation serves as a critical mediator of inflammation and tissue injury. Elevated IRF3 phosphorylation levels observed in the livers of patients with PBC and Primary Sclerosing Cholangitis (PSC) reveal that bile acid–induced IRF3 activation mediates cell death, inflammatory responses, and fibrosis, highlighting the pivotal role of innate immune signaling in disease pathology (71). The immune cell landscape also undergoes alterations in PBC. ILC subsets, specifically ILC1s and ILC3s in both patients and murine models, are implicated in the disease process, potentially contributing to inflammatory responses and biliary tract fibrosis (72).

Bile acids themselves function as modulators of inflammation. The roles of bile acids and their cognate receptors underscore their influence on immune responses in autoimmune liver diseases (73). This connection implies that bile acid–mediated signaling pathways may intersect with inflammatory cascades, further modulating innate immunity in PBC. The microbial milieu within the biliary system likewise influences inflammatory responses. Altered biliary microbial patterns correlate with disease progression and reduced transplant-free survival in PSC, suggesting microbial factors modulate innate immune activation and inflammation in cholangiopathies (74).

Additionally, systemic inflammatory cytokines and their mediators have been investigated for causal roles (**Table 1**). Mendelian randomization analyses demonstrate that circulating inflammatory cytokines mediate the relationship between plasma metabolites and bile duct or gallbladder calculus formation (91), collectively illustrating the integral contribution of inflammatory mediators to disease pathogenesis and progression.

**Table 1:** Primary cytokines associated with cholangiocytes and their functional roles in PBC.

Cytokines	Function Description
IL-1β	IL-1β upregulates the expression of intercellular adhesion molecules, antigen-presenting molecules, and IL-6 in cultured cholangiocytes and induces Th17 cell differentiation in conjunction with IL-6 (75).
IL-2	Low-dose IL-2 can significantly improve liver biochemistry and pathology by reversing the imbalance of Th17 and Treg cells (23, 76)
IL-6	IL-6 mediates the proliferation of cultured cholangiocytes and, in conjunction with IL-1ß, promotes Th17 cell differentiation (77, 78).
IL-10	The immunomodulatory effect of IL-10 as an immunosuppressive and anti-inflammatory cytokine as a role of IL-10 in fibrosis (79–81).
IL-12	IL-12 enhances T cell activation, leading to IFN-y-mediated cholangiocyte injury via Th1 responses in PBC (82).
IL-17	The frequency of IL-17 (+) lymphocyte infiltration in liver tissues of patients with PBC and those with other liver dysfunction increases. IL-17A promotes fibrosis related to PBC (47, 81, 83).
IL-21	IL-21 is a member of the common gamma-chain cytokine family and exacerbated liver fibrosis in mice with autoimmune cholangitis (84, 85).
IL-23	The persistence of IL-12/ Th1-mediated immunopathology in PBC through the IL-23/Th17 pathway (86).
TNF-α	TNF-α upregulates the expression of intercellular adhesion molecules and antigen-presenting molecules in cultured cholangiocytes and disrupts the barrier function of the tight junction (30, 87).
IFN-γ	IFN-γ upregulates the expression of intercellular adhesion molecules and antigen-presenting molecules, downregulates PPAR-γ expression in cultured cholangiocytes, and disrupts the barrier function of the tight junction (75, 87).
TGF-β	NUDTI-dependent DNA damage resistance enhances CD8+ T cells in vitro through the PARP1-TGF ßR axis (62, 88)
MMP-3	The increase in MMP-3 concentration is positively correlated with various clinical and immunological parameters of PBC as well as advanced liver fibrosis (89).
ERα	ERα activation led to mitochondrial damage, apoptosis, and upregulation of ERα and PDC-E2 expression (90).

This table delineates key cytokines implicated in the pathogenesis of PBC, detailing their effects on cholangiocytes and their contributions to disease mechanisms including inflammation, immune cell differentiation, fibrosis, and disruption of biliary barrier integrity. Each cytokine entry is accompanied by a description of its primary function(s), as established by current evidence from vivo or vitro investigations. Superscript numerals denote corresponding references.

CD, a cluster of differentiation; IFN, interferon; MMP, matrix metalloproteinase; PBC, primary biliary cholangitis; PPAR, peroxisome proliferator-activated receptor; TGF, transforming growth factor; Th, T helper; TNF, tumor necrosis factor. Cytokines expressed by cholangiocytes from Refs (30, 75, 86, 87, 89).

### 3.4 Biliary microbiota

The biliary microbiome constitutes a complex microbial community inhabiting the biliary tract. Dysbiosis—an imbalance within this ecosystem. Specifically, patients with PBC exhibit compositional alterations in their biliary microbiota, which may drive immune dysregulation and disease progression (92, 93). Certain bacterial genera, including *Enterococcus* and *Fusobacteria*, demonstrate significant correlations with PBC severity. The detection of *Enterococcus* in bile samples is associated with heightened risks of disease advancement, suggesting that specific microbial populations exacerbate inflammatory cascades within the biliary tract (41, 94).

Biliary epithelial cells regulate local immune responses through secretory IgA expression and chemokine receptor modulation, while bile acids confer cytoprotection via TGR5 receptor activation (95). Conversely, secondary bile acids generated by microbial metabolism exhibit concentration-dependent and microbiota-contextual effects on cholangiocytes, ranging from protective to detrimental outcomes (41, 96). The interplay between biliary microbiota and inflammation involves multifactorial mechanisms. Dysbiosis may promote intestinal barrier dysfunction, facilitating bacterial translocation and metabolite influx into the biliary system. This process can incite aberrant immune activation and cholangiocyte injury (93, 97). Distinct microbial signatures within the bile of PBC patients correlate with disease duration and severity. Elevated microbial richness and enrichment of specific taxa are linked to advanced fibrotic stages (98).

Given the emerging role of biliary dysbiosis in PBC, microbiome-targeted interventions represent a promising therapeutic strategy. Approaches including probiotics, prebiotics, and fecal microbiota transplantation aim to restore microbial homeostasis and enhance biliary immune function (99, 100). Pharmacological agents modulating bile acid metabolism and cholangiocyte signaling pathways also hold therapeutic potential as PPAR agonists (23, 32).

## 4 Diagnosis and treatment strategies for innate biliary immunity

### 4.1 The standard of care

**Figure 3:**
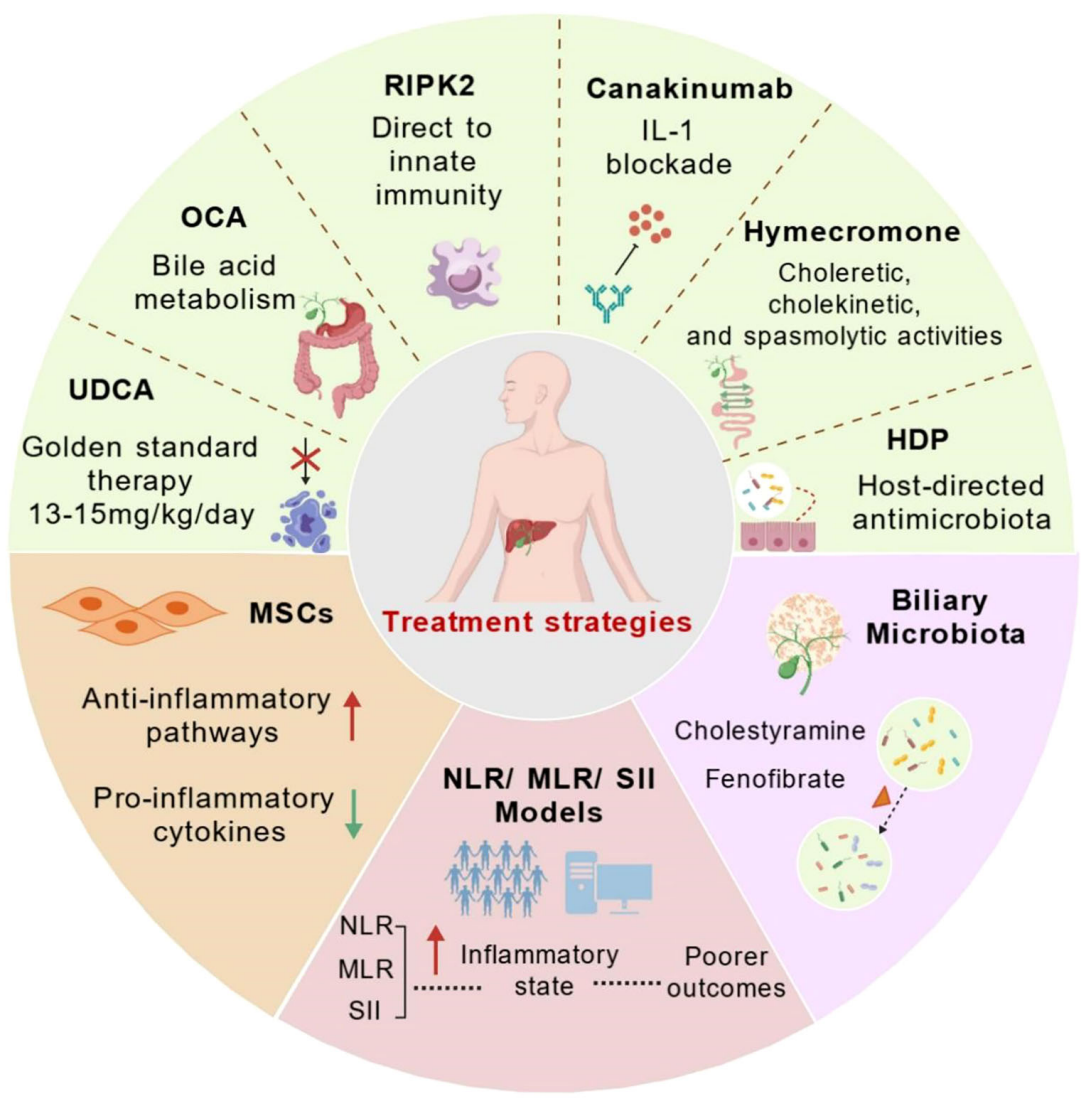
Therapeutic approaches in primary biliary cholangitis. The figure summarizes current and emerging approaches to modulate innate immune responses and inflammation in PBC. Schematic overview of current standard of care (UDCA and OCA) and emerging therapeutic strategies (RIPK2 inhibition, IL-1 blockade, TIM-3 modulation, MSCs, Biliary Microbiota therapy and novel prognostic markers - NLR/MLR/SII), highlighting potential molecular targets and pathways in PBC. Established therapies: UDCA (first-line) and OCA (obeticholic acid, FXR agonist) primarily target bile acid metabolism. Investigational and adjunct therapies: PPAR-α agonists (e.g., fenofibrate) and IL-1 blockade (e.g., canakinumab). Exploratory strategies: RIPK2 inhibition (innate immunity), Hymecromone (choleretic effects), MSCs therapy(anti-inflammatory), and HDPs (biliary microbiota modulation). Systemic inflammation indices NLR, MLR, SII serve as prognostic biomarkers, with elevated levels correlating with a pro-inflammatory state and poorer outcomes, guiding personalized treatment decisions. Created by BioGDP.com (29). UDCA, ursodeoxycholic acid; OCA, Obeticholic Acid; RIPK2 inhibitors, immunomodulation; Canakinumab, IL-1 blockade; MSCs, anti-inflammatory modulation; HDPs, Host-Directed Therapies; Cholestyramine, bile acid sequestrant; Fenofibrate, PPAR-α agonist; NLR, Neutrophil-to-Lymphocyte Ratio; MLR, Monocyte-to-Lymphocyte Ratio; SII, Systemic Immune-Inflammation Index.

UDCA persists as the cornerstone of PBC management, attenuating cholestatic injury through pleiotropic mechanisms (32, 100). Although its cytoprotective effects on cholangiocytes against bile acid toxicity are well-established, the precise molecular pathways—particularly its interactions with bile acid transporters and nuclear receptors—require further elucidation. This knowledge gap holds clinical significance: approximately 40% of patients exhibit suboptimal biochemical responses to UDCA, necessitating second-line therapies (41). OCA, a potent FXR agonist, targets this therapeutic gap by reprogramming bile acid homeostasis. Its efficacy derives from the transcriptional upregulation of efflux transporters (BSEP, MRP2/3, MDR3), thereby reducing hepatocellular bile acid retention and apoptosis (43, 101). However, the dose-dependent pruritus of OCA and heterogeneous treatment responses underscore the necessity for personalized FXR agonist selection based on patient genetics and disease phenotype. The autoimmune pathogenesis of PBC positions innate immunity regulators as promising therapeutic targets, as illustrated in **Figure 3**.

### 4.2 Emerging experimental strategies targeting innate immunity

Receptor Interacting Serine/Threonine Kinase 2 (RIPK2) functions as a critical signaling hub within the NOD-mediated inflammatory cascade (102). Pharmacological inhibition of RIPK2 may disrupt the self-perpetuating cycle of biliary epithelial cell damage and cytokine release- a strategy potentially synergistic with bile acid-directed therapies. Similarly, interleukin-1 blockade (Canakinumab) represents a rational therapeutic approach to attenuate inflammasome activation in patients refractory to UDCA/OCA (103). Its established efficacy in autoinflammatory disorders such as Still’s disease suggests applicability in PBC patients exhibiting prominent serological inflammation (elevated CRP/SAA). Nevertheless, strategic stratification of patients is essential: IL-1 antagonists may benefit individuals with dominant innate immune activation, whereas RIPK2 inhibitors might target those with NOD pathway dysregulation. Non-immunosuppressive adjuncts play vital roles in symptom control and mucosal protection. Hymecromone exemplifies this approach—its triple mechanisms of action (choleretic, cholekinetic, spasmolytic) alleviate biliary pain without inducing gallbladder contraction, rendering it uniquely suited for biliary dyskinesia (104, 105). Concurrently, the gut-liver axis emerges as a therapeutic target. Host defense peptides (HDPs), induced by dietary components, enhance mucosal barrier integrity and exert selective antimicrobial effects without exacerbating inflammation (105). This host-directed strategy could counteract dysbiosis-associated disease progression, particularly given evidence that UDCA modulates gut microbiota composition- a potential biomarker for treatment response (106).

The therapeutic landscape of PBC is poised for transformation through innovative strategies extending beyond conventional approaches. Drug repurposing spearheads this evolution, with network pharmacology identifying promising candidates—including IL-1, EGFR, and TNF-α inhibitors, branched-chain amino acids, and curcumin—tailored to distinct PBC endotypes (107). These agents offer accelerated translational potential due to established safety profiles, enabling rapid deployment for phenotype-specific interventions. Building on cross-disciplinary insights, oncology-inspired therapeutic synergies present compelling paradigms. The efficacy of CDK4/6 inhibitors combined with cytotoxic chemotherapy in biliary tract cancers demonstrates how autophagy blockade overcomes treatment resistance (108). While direct applicability to PBC requires validation, this mechanistic approach—particularly triple therapy regimens targeting autophagic flux—could inform combinational strategies for advanced, treatment-refractory PBC.

Furthermore, advanced delivery systems are redefining precision targeting. Engineered exosomes exemplify this frontier, functioning as modular platforms for cholangiocyte-directed immunomodulation (109). Their capacity to deliver bespoke cargo (e.g., miRNA silencing RIPK2 or anti-inflammatory cytokines) could overcome limitations inherent to systemic drug administration, enabling site-specific pathway modulation with reduced off-target effects. Additionally, immunogenic microenvironment reprogramming—inspired by oncology’s “cold-to-hot” tumor conversion strategies—holds untapped potential. Activating Double-stranded RNA (dsRNA) sensors within fibrotic hepatic niches may reverse immunological anergy, priming PBC microenvironments for enhanced responsiveness to checkpoint inhibitors (110). This approach could synergize with existing immunomodulators to interrupt cycles of autoimmune-driven fibrosis.

### 4.3 Stem cell therapy drugs

Mesenchymal stem cells (MSCs) represent a promising therapeutic strategy for PBC owing to their immunomodulatory properties, capacity for multilineage differentiation, and potential to promote tissue repair (111, 112). MSCs modulate immune responses by suppressing pro-inflammatory cytokine production and augmenting anti-inflammatory pathways, thereby potentially restoring immune homeostasis in PBC patients (41, 111). MSC therapy may mitigate the effects of trained immunity—a phenomenon wherein innate immune cells exhibit hyperresponsiveness to secondary stimuli that may otherwise exacerbate pathologies such as stroke (41, 112).

However, the clinical translation of MSC therapy for PBC remains nascent, with ongoing research focused on optimizing therapeutic protocols. Clinical trials have demonstrated promising efficacy, indicating MSC administration improves hepatic function and attenuates inflammation in autoimmune liver diseases (112–114). Nevertheless, clinical trials in autoimmune liver diseases are ongoing, with results awaited (NCT02997878). The challenges persist, including the necessity for standardized methodologies for MSC isolation, expansion, and delivery, alongside concerns regarding long-term therapeutic safety and efficacy (113, 114).

Furthermore, integrating MSC therapy with established treatments may enhance clinical outcomes. Combining MSC therapy with immunomodulatory agents could yield synergistic effects, advancing overall PBC management (115, 116). Recent advances in stem cell membrane-camouflaged nanoparticles demonstrate potential for targeted delivery to inflamed tissues, offering a particularly advantageous approach for PBC treatment (117).

### 4.4 Prognostic model of NLR, MLR, and SII

Neutrophil-to-Lymphocyte Ratio (NLR) and Monocyte-to-Lymphocyte Ratio (MLR) are readily available and cost-effective markers of systemic inflammation (118–120). The potential utility of NLR and MLR as predictors of treatment response to novel therapies for PBC. However, these biomarkers have not yet been validated for routine clinical application and are not incorporated into current international management guidelines. NLR reflects the balance between innate (neutrophils) and adaptive (lymphocytes) immune responses (121). Elevated NLR indicates a heightened inflammatory state, often associated with poorer outcomes in various diseases (120). Similarly, MLR reflects the proportion of monocytes relative to lymphocytes, providing another dimension of immune system activity (121, 122).

Several studies have demonstrated the prognostic value of NLR and MLR in various diseases, including autoimmune conditions and cancers (119–130). In seropositive autoimmune encephalitis, a high NLR was associated with a higher likelihood of first-line treatment failure (129). In advanced gastric and colorectal cancers, lower MLR was associated with prolonged progression-free survival and overall survival (120). Higher NLR has been correlated with poor prognosis in several cancers, as well as being a reliable marker of inflammation, infection and sepsis (121, 129). High NLR levels independently associated with poor prognosis in heart failure (129). Therefore, NLR and MLR can serve as indicators of disease severity and predictors of treatment response and survival. A study found the maximal NLR had the best predictive value for in-hospital and 30-day mortality in ICU patients with CAD and CKD (128). Meanwhile, the total cholesterol, ALP, and NLR were the three independent risk factors associated with early biochemical nonresponse to UDCA treatment (129).

While specific studies focusing on NLR and MLR as prognostic markers in PBC are still emerging, the existing literature provides a strong rationale for their potential utility. Combining NLR and MLR with other inflammatory markers, such as the Systemic Immune-inflammation Index (SII) (122, 123, 125, 126), to improve prognostic accuracy (38).

### 4.5 Biliary microbiota therapy

Dysbiosis, or an imbalance in the gut microbiota, has been implicated in the pathogenesis of PBC. For instance, alterations in the gut microbiome have been associated with the severity of liver disease and the response to treatment. Research indicates that specific microbial taxa may influence the immune response and contribute to the autoimmune processes observed in PBC patients (131). Furthermore, the salivary microbiota of PBC patients exhibited significant differences, suggesting that oral microbiota may play a role in the disease’s pathogenesis (132).

Consequently, given the emerging evidence of the gut-liver axis’s role in PBC, several therapeutic strategies are being explored to manipulate the biliary microbiota. One promising approach involves the use of bile acid sequestrants, such as cholestyramine, which have been shown to alter the gut microbiome and improve cholestatic symptoms in PBC patients (133). Additionally, the use of fenofibrate as a second-line therapy for patients with inadequate responses to UDCA has shown promise. Studies have demonstrated that fenofibrate can improve liver biochemistry and histological features in PBC patients, suggesting that it may also exert beneficial effects on the gut microbiota (134). The combination of UDCA and fenofibrate has been associated with improved biochemical responses, indicating a synergistic effect that may be mediated by changes in the gut microbiome (135). The exploration of biliary microbiota therapy in PBC is still in its infancy. Moreover, clinical trials investigating the efficacy of probiotics, prebiotics, and other microbiota-targeted therapies in PBC patients are warranted (136). Beyond cholestatic pathologies, Hypoxia-inducible Factors (HIFs) exhibit immunosuppressive properties in hepatic malignancies by fostering pro-tumorigenic microenvironments, positioning them as therapeutic targets in liver cancer management (137).

### 4.6 CAR-T therapy

Originally developed for hematological malignancies, Chimeric Antigen Receptor (CAR)-T cell therapy has recently demonstrated sustained, profound depletion of autoreactive B cells in autoimmune diseases, exhibiting promising safety and efficacy profiles (138). This transition from oncology to autoimmunity represents a transformative advancement in therapeutic strategy (139). Currently, CAR-T therapy is under investigation across 372 institutions in 40 countries/regions for various autoimmune conditions, including Systemic Lupus Erythematosus (SLE) and multiple sclerosis (140). The development of novel CAR architectures, such as fourth-generation constructs, continues to enhance therapeutic potential (141, 142).

However, CAR-T therapy entails significant risks, including Cytokine Release Syndrome (CRS), neurotoxicity, and organ-specific toxicities. These adverse events are influenced by inflammatory microenvironments, limitations inherent in CAR design, and systemic immune disruption (143). Safety optimization may be achievable through innovative strategies—e.g., logic-gated CAR systems or alternative cellular carriers—coupled with rigorous clinical monitoring. Future efforts should prioritize generating disease-specific clinical evidence and developing adaptable CAR designs to facilitate broader, safer application.

Although CAR-T expansion into autoimmunity offers new therapeutic potential for refractory cases (144–147), its adverse effects present distinctive challenges within autoimmune pathological contexts (138, 139). For instance, inflammatory microenvironments can impair CAR-T persistence and exacerbate toxicity (140). Disease-specific toxicities, such as renal dysfunction or cutaneous manifestations, have also been reported, particularly with CD19-directed CAR-T therapy in SLE (146). Research characterizing toxicity profiles is evolving rapidly. Critical future directions include developing allogeneic CAR-T products to circumvent limitations associated with autologous approaches and extending CAR technology to alternative effector cells (e.g., CAR-macrophages or CAR-Natural Killer (NK) cells) to improve safety and efficacy (148, 149). Integration of precision medicine methodologies may facilitate enhanced risk stratification and personalized monitoring protocols (150). Therefore, Refined CAR designs and systematic real-world evidence are essential for safe, effective translation from oncology to autoimmunity. It must be emphasized that the application of CAR-T therapy in PBC remains speculative, there is no clinical evidence currently supports its potential efficacy in this condition.

## 5 Conclusions and future perspectives

Innate immune pathways—including TIM3/Gal9, NF-κB, and cGAS-STING—emerge as promising intervention points for biliary tract disorders, given their central role in stone-associated inflammatory cascades (151–154).

The innate immune system constitutes a pivotal pathogenic mechanism in PBC. Dysregulation of innate immune responses can activate autoreactive T and B lymphocytes, contributing to autoimmunity (155). Recent scientific advances demonstrate that apoptosis of biliary epithelial cells releases auto-antigens, which subsequently activate the immune system and disrupt immune tolerance (41, 156). Furthermore, the gut-liver axis is implicated in PBC pathogenesis, with gut microbiota and bile acids influencing immune responses and disease progression (75).

Although strategies targeting innate immunity—such as RIPK2 inhibition, IL-1 blockade, TIM-3 modulation, and MSCs therapy—hold therapeutic promise, they remain largely at the experimental stage and require further validation before clinical translation. Furthermore, novel prognostic inflammatory markers including NLR and MLR have been shown to reflect systemic inflammation and correlate with disease progression. In parallel, deeper investigation into mechanisms such as the gut–biliary–immune axis and trained immunity is ongoing. However, these approaches currently lack sufficient clinical evaluation, and their application remains speculative. Firstly, the complexity of the immune response in PBC complicates the identification of specific intervention targets. For instance, while Tregs are crucial for maintaining immune tolerance, their therapeutic application in autoimmune liver diseases has yielded inconsistent outcomes (156). While the role of autoimmunity in the pathogenesis of PBC is well established, immunomodulatory therapies (including biologics) effective in other autoimmune diseases may be ineffective in PBC (157). Furthermore, existing experimental models of PBC inadequately recapitulate the key immunopathological features, progression kinetics, and heterogeneity observed in human (158, 159). Consequently, immunomodulatory strategies developed based on model data frequently fail to achieve anticipated efficacy in the clinical trials of human. Moreover, the protracted progression characteristic of PBC presents substantial challenges for clinical trial design. Studies often rely on surrogate endpoints, and the implementation of long-term placebo-controlled trials is problematic. These limitations impede the definitive confirmation of benefits on hard clinical endpoints. Additionally, trial durations may be insufficient to demonstrate meaningful therapeutic effects (28, 160).

Critically, a fundamental limitation of failed therapies lies in their inability to effectively target core liver/bile duct-specific immunopathological mechanisms. Specifically, these include pathogenic CD8^+^T cells, dysregulated IL-15 signaling, and tissue-resident memory T cells. Many therapeutic approaches, however, induce generalized immunosuppression without effectively intervening in the early stages of the autoimmune cascade (28).

Additionally, precise modulation of the immune response is critical, as overactivation can exacerbate hepatic damage. Advancements in immunotherapy offer promising avenues for PBC treatment. The therapeutic application of MSCs has emerged as a potential strategy due to their immunomodulatory properties and capacity to facilitate tissue regeneration (114). Concurrently, biopolymer immune implants designed to sequentially activate innate and adaptive immunity show promise in other contexts, suggesting potential applicability in PBC (161). Moreover, exploration of the gut microbiota-bile acid-immunity network provides a novel perspective for therapeutic strategies. Targeting immune factors associated with gut microbiota dysbiosis and bile acid imbalance may yield breakthroughs in PBC management (75, 120). Converging research underscores the critical regulatory role of the STING pathway in innate immunity, revealing novel therapeutic opportunities (162). Genetic associations between PBC and extrahepatic autoimmune disorders (e.g., inflammatory bowel disease) may indicate shared pathogenic pathways, potentially informing the development of targeted interventions (163, 164).

Collectively, the biliary innate immune system in PBC critically regulates disease progression through intricate cellular and molecular mechanisms. Damage to bile duct epithelial cells, release of inflammatory mediators, and immune cell imbalance collectively drive autoimmune responses and fibrotic processes. Technological innovations, including T Cell Receptor (TCR) sequencing and Chimeric Antigen Receptor (CAR) platforms, demonstrate potential for engineering personalized immunotherapies addressing PBC-specific immune dysfunction (141, 145).

Emerging therapeutic strategies—including targeting innate immune signaling pathways, modulating the gut microbiota, and applying stem cell therapy—provide novel approaches for PBC management. These findings elucidate key immunological nodes in PBC pathogenesis, thereby establishing a foundation for developing precise therapeutic strategies. Nevertheless, these methods presently do not have adequate clinical assessment, and their use is still hypothetical. Consequently, thorough mechanistic studies and stringent treatment burden evaluation are crucial to promote these strategies for clinical application. Future research should prioritize elucidating the gut-liver axis, bile acid metabolism and CAR-T cells, which are anticipated to yield more effective PBC treatment options.
